# Analysis of Presurgical Language in Children with Posterior Fossa Tumours Relative to Postoperative Speech Outcomes: Findings from the European CMS Study

**DOI:** 10.1007/s12311-026-01987-3

**Published:** 2026-04-10

**Authors:** Aliene Reinders, Cheyenne Svaldi, Annet Kingma, Jonathan Kjær Grønbæk, Ditte Boeg Thomsen, Karin Persson, René Mathiasen, Christine Dahl, Andrea Carai, Bianca Andreozzi, Angela Mastronuzzi, Barry Pizer, Colin Thorbinson, Kristian Aquilina, Eelco Hoving, Marianne Juhler, Roel Jonkers, Vânia de Aguiar

**Affiliations:** 1 Center for Language and Cognition Groningen (CLCG), University of Groningen, Groningen, The Netherlands; 2 Research School of Behavioural and Cognitive Neurosciences (BCN), University of Groningen, Groningen, The Netherlands; 3 Division of Psychology and Language Sciences, University College London, London, UK; 4 Department of Pediatrics/Pediatric Oncology, University Medical Center Groningen, Groningen, The Netherlands; 5 Department of Paediatric and Adolescent Medicine, Copenhagen University Hospital Rigshospitalet, Copenhagen, Denmark; 6 Department of Nordic Studies and Linguistics, University of Copenhagen, Copenhagen, Denmark; 7 Department of Health Sciences, Lund University, Lund, Sweden; 8 Neurosurgery Unit, Bambino Gesù Children’s Hospital, Rome, Italy; 9 Department of Hematology/oncology, Cell Therapy, Gene Therapies and Hematopoietic Transplant, IRCCS Bambino Gesù Children’s Hospital, Rome, Italy; 10 Department of Life Sciences and Public Health, Università Cattolica del Sacro Cuore, Milan, Italy; 11 University of Liverpool, Liverpool, UK; 12 Department of Oncology, Alder Hey Children’s NHS Foundation Trust, Liverpool, UK; 13 Department of Neurosurgery, Great Ormond Street Hospital, London, UK; 14 Oncological Pediatric Neurosurgery, Princess Maxima Center for Pediatric Oncology, Utrecht, The Netherlands; 15 Department of Neurosurgery, Aarhus University Hospital, Aarhus, Denmark; 16 Department of Radiation Oncology, University Medical Center Groningen, Groningen, The Netherlands

**Keywords:** Cerebellar mutism syndrome, Mutism, Posterior fossa syndrome, Infratentorial neoplasms, Preoperative language impairment, Language disorders

## Abstract

Posterior Fossa Syndrome is a common complication in children following posterior fossa tumour surgery, typically marked by transient postoperative speech impairment (POSI; i.e., mutism or reduced speech). Previous studies suggest that children who develop POSI show different language profiles postoperatively compared to those who do not. It remains unclear to what extent these language difficulties exist preoperatively and whether preoperative *language* difficulties are related to postoperative *speech* status. This study provides the first comprehensive analysis of preoperative language samples, using data from the European study of Cerebellar Mutism Syndrome. Patients with and without POSI were compared to identify preoperative language characteristics that may be associated with POSI. Preoperative language samples of 34 patients aged 3–16 years were analysed (16 developed POSI; 18 did not). We compared global sample characteristics and language performance across four levels: the semantic, lexical, morphosyntactic, and phonological level. No significant preoperative language differences were found between the groups for the four levels of language processing (all *p*-values > 0.137). Children who developed POSI produced more unintelligible speech preoperatively (β = -14.455, *p* = .024), with better intelligibility related to older age (age×group: β = 0.152, *p* = .007), only for the group with POSI. While the main focus of this study was on language, these findings suggest that risk factors for POSI within the domain of verbal output may lie more in preoperative speech. A comprehensive analysis of preoperative speech may provide valuable insight into speech characteristics potentially related to POSI.

**Supplementary Information:**

The online version contains supplementary material available at 10.1007/s12311-026-01987-3.

## Introduction

Approximately half of the brain tumours in children occur in the posterior fossa [1]. Treatment for Posterior Fossa Tumours (PFTs) generally entails neurosurgical resection, which is often followed by chemo- and radiotherapy [2]. Posterior Fossa Syndrome (PFS), also referred to as Cerebellar Mutism Syndrome (CMS), is a common complication following PFT surgery in children [3]. It typically arises within days after the neurosurgical resection and affects around 24–34% of patients [4–7]. The most defining symptom of PFS is transient mutism (PFS1; [8]) or severely reduced speech (PFS2), typically lasting from a couple of days to 6 months [9], with an average duration of about 8 weeks [10]. Mutism and severely reduced speech are also jointly referred to as postoperative speech impairment (POSI) [5]. PFS is further characterised by emotional lability, hypotonia, and a wide range of motor and cognitive deficits [11]. Although the mutism or reduced speech is transient, speech problems can persist later in life [12]. Importantly, long-term deficits associated with postoperative mutism or reduced speech extend beyond *motor speech* production, and affected children exhibit broad neurocognitive vulnerabilities, including in the domain of *language* [13], reflecting the cognitive system underlying the representation and processing of linguistic information. This highlights the importance of identifying early (cognitive) markers that may be associated with the development of postoperative mutism or reduced speech.

While some studies have examined preoperative language functioning in this population, these have typically relied on broad or composite language measures rather than providing an in-depth profile of language abilities across linguistic processing levels [14, 15]. As a result, potential presurgical differences in language functioning between children who go on to develop postoperative mutism or reduced speech compared to those with habitual speech remain insufficiently characterised. In this study, we therefore perform a comprehensive analysis of narrative language samples of children diagnosed with a PFT before they undergo neurosurgical resection. Their language performance will be related to their postoperative speech status (mutism or reduced speech vs. habitual speech), to identify if there are language characteristics that may be related to the emergence of mutism or reduced speech.

### Speech and Language after Mutism or Reduced Speech

When mutism or reduced speech subsides, patients will often present with motor speech disorders, a pattern collectively termed mutism with subsequent dysarthria [16]. They may also have more impairments in the initiation of voluntary movement, including speech initiation [17], termed adynamic speech [18]. However, long-term impairments occur in both speech and language in paediatric PFT survivors regardless of whether they experienced mutism or reduced speech or not [13, 19, 20]. Nonetheless, several studies suggest that there may be differences in either the nature or the severity of motor and cognitive impairments observed, and specifically in speech and language abilities of patients who experienced mutism or reduced speech compared to patients who did not [16–18, 21].

Patients who experienced mutism were reported to have poorer outcomes in verbal comprehension, receptive and expressive language, verbal memory, and verbal fluency [19]. Additionally, the presence of mutism or reduced speech was found to be related to slower reading pace [22] and poorer verbal learning [23]. In Svaldi et al. [13], children who experienced mutism were found to show language impairments predominantly in the morphosyntactic and semantic domains of language, while children who did not develop mutism showed a wider spread in language impairments across all language domains. However, in a recent study investigating postoperative word-finding abilities in a large sample, Persson et al. [20] did not find a relation between postoperative speech impairment (POSI, consisting of mutism or reduced speech) and poorer postoperative word-finding abilities. Still, all children who had experienced a complete absence of speech exhibited a postoperative decline in word finding.

Studies examining language differences between patients with or without mutism or reduced speech included either small participant numbers [13], or focused on a specific aspect of language [20]. Furthermore, most studies did not account for preoperative language impairment (with the exception of [20]). This is important, as it may be that preoperative language characteristics can be linked to the emergence of mutism or reduced speech [14, 15].

### Risk Factors for Mutism or Reduced Speech

Extensive research has been done on the risk factors related to the emergence of mutism or reduced speech, see [24] for an overview. Several studies show that the incidence of mutism or reduced speech decreases with age [5, 6, 25], with rare occurrence in adults [26]. İldan et al. [27] proposed that the higher incidence of mutism in younger children could be related to the incomplete maturation of the brain that renders younger children more prone to developing the complication. Additionally, some tumour locations are associated with higher risk of mutism or reduced speech, such as vermal and midline tumours [4, 5, 28] and brainstem tumours [7]. Cerebellar hemisphere tumours, on the other hand, generate a lower risk [5, 7]. Concerning tumour type, patients with high-grade medulloblastomas have consistently been reported to develop mutism or reduced speech more often than those with low-grade astrocytomas [4, 5].

Preoperative risk factors in the domain of language are underexplored, as previous research primarily focuses on differential postoperative language outcomes in patients with mutism or reduced speech. Nonetheless, Di Rocco et al. [15] found that children in their sample without preoperative language problems did not develop mutism, while all children who developed mutism presented with preoperative language problems, characterised by a shorter Mean Length of Utterance (MLU) and problems with verbal fluency and lexical naming. Bianchi et al. [14] extended the findings of Di Rocco et al. by enlarging the patient cohort, showing that 20 out of 70 patients with PFTs who developed mutism presented with preoperative language impairments. Persson et al. [29] found that patients with PFTs experience word finding difficulties before neurosurgical resection, characterised by slow and/or inaccurate word finding, but they did not examine the relationship between preoperative word finding difficulties and the emergence of mutism or reduced speech. Research into the preoperative language abilities of children with PFTs is thus limited, and no research has comprehensively compared the language profiles of children who do and do not experience mutism or reduced speech across different levels of language processing.

### Identifying Language Impairments Through Connected Language

The existing literature on language disorders in child survivors of PFTs suggests that these may affect all language domains (e.g., phonology, morphosyntax, lexical and semantic knowledge, pragmatics) and may present themselves in variable combinations and severity across children [13, 30]. Preoperative language abilities should thus be evaluated comprehensively in every patient, including all language domains. A highly productive approach to evaluate multiple domains of language is through the evaluation of language samples, either elicited in conversations/interactions, picture descriptions, or through the (re)telling of narratives [13, 31, 32]. This approach has been used previously in children with PFTs, focusing on the macro- and microstructural aspects of language after neurosurgical resection and revealing differences from healthy controls [13, 33] as well as differences between subgroups [34].

This approach combines the use of standard measures (e.g., MLU) and the critical variable approach by Shallice [35], similarly to Svaldi et al. [13], see also the Supplementary Material for a detailed description of this approach. In the critical variable approach, Shallice describes that properties of words (i.e., psycholinguistic properties) can impact language performance, and that these properties, which reflect functioning of specific levels of language processing, can reveal impairments at their respective levels.

### Levels of Language Processing

Across models of language processing (e.g. [36–38]), it is commonly agreed that conveying a message (e.g., ‘The cat meows’) through spoken language requires processing at several levels of language. At the semantic level, conceptual information related to the meanings of words is being stored and retrieved (e.g., the knowledge that a cat meows; [38]). At the lexical level, the word forms which make up our mental dictionary, or lexicon, are stored and retrieved [38]. The morphosyntactic level relates to the internal structure of words (morphology) and sentences (syntax) [39]. Morphological rules govern the internal structure of words and establish a relation to other units in the syntactic structure (e.g., the addition of ‘s’ to the verb-stem ‘meow’, to match the third person singular of the subject). Syntax consists of grammatical information such as word classes (e.g., ‘cat’ is a noun, ‘meowing’ is a verb), as well as rules concerning sentence structure (e.g., the subject ‘cat’ must come before the verb ‘meows’). Furthermore, at the phonological level, segmental phonological information is retrieved. This entails information of individual speech sounds, which need to be ordered correctly and stored in phonological short-term memory in preparation for and during speech [38]. Breakdowns at each of these levels can lead to characteristic patterns of errors (see [40]).

For each of these levels, there are linguistic variables which may be extracted from narrative language to study their functioning. For example, semantic representations may be easier to retrieve depending on how easily a concept evokes a mental image (imageability) [41] or how concrete/abstract it is [42], so patients with semantic disorders may be biased to use the words which are highly imageable and concrete (e.g. [13, 43]), . Other variables that tap into the semantic system are familiarity [44] or instrumentality of verbs [45]. At the lexical level, vocabulary size can be estimated with the ratio of different to total words in a sample (Type-token ratio, TTR), although this measure is known to be sensitive to sample length [46]. Alternatively, lexical properties, such as lexical accuracy, corpus-based word frequency [47], or word age of acquisition [48] may be studied. Morphosyntactic ability may be assessed with measures such as the Mean Length of Utterances (e.g. [49]), , as well as grammatical accuracy [50], and proportion of finite verbs (i.e., inflected verbs such as ‘walks’, as opposed to ‘walk’), with the latter two showing impairment in children with PFTs [13]. Other word properties such as verb transitivity, unaccusativity, and regularity of inflectional paradigms, can also be used to detect atypical verb usage in populations with language impairment (e.g. [51, 52]), . At the phonological level, impairments may be revealed by phonological errors [53], or a bias to produce less complex articulatory patterns (e.g., ‘string’ vs. ‘sing’) [54, 55]. Furthermore, a tendency to produce short words may be indicative of phonological short-term memory difficulties [56]. While most of these variables were included in the work by Svaldi et al. [13] on postoperative language, such a comprehensive analysis of narrative language has not been reported in studies concerning preoperative language abilities of PFT patients.

### Current Study

In summary, postoperatively, in addition to speech difficulties, there may be language characteristics that distinguish children who have experienced postoperative speech impairment from those who have not. Research into language in the preoperative stage is limited but suggests that linguistic differences between patients with mutism or reduced speech compared to those with habitual speech might already be present before neurosurgical resection. However, knowledge on the exact nature of these preoperative impairments and what language characteristics are related to the emergence of mutism or reduced speech remains unclear. This study will be the first to extensively analyse the preoperative language samples of patients who underwent neurosurgical resection for a PFT and did or did not develop postoperative speech impairment. Language samples of 34 patients will be analysed, replicating and expanding on language analysis procedures used by Svaldi et al. [13]. By taking postoperative speech status as the grouping factor, we aim to examine whether differences between the groups are present in cognitive domains beyond speech, specifically language functioning, prior to surgery. Namely, we investigate whether preoperative semantic, lexical, morphosyntactic, and phonological characteristics may be related to the emergence of postoperative mutism or reduced speech. Strengthening our understanding of preoperative language abilities as a risk factor for the emergence of this complication will help predict postoperative outcomes and allow better preparation of patients and their parents regarding potential difficulties that might await them after surgery.

## Method

### Participants

Data from children who underwent surgery for a PFT were retrieved through a database that is part of the prospective European CMS Study [57]. This is a multicentre prospective study in which approximately 40 treatment centres across 13 European countries collect data on patients treated for a posterior fossa tumour at several stages of the treatment trajectory. The study was registered on November 24, 2014, with ClinicalTrials.gov (registration number: NCT02300766). Within the European CMS Study, postoperative mutism or reduced speech is referred to as Postoperative Speech Impairment (POSI), a term we adopt in the current study. Between 2013 and 2024, 794 patients participated in the study. We included patients from treatment centres in The Netherlands, The United Kingdom and Italy, who were assessed in Dutch, English and Italian, respectively. As the number of children undergoing surgery for a PFT within individual countries is limited, inclusion of participants across multiple language backgrounds enabled the investigation of preoperative language differences between children with and without POSI in a sufficiently large cohort. Criteria for inclusion in the current study were: (1) the availability of a language sample collected before the first neurosurgical resection of a PFT, (2) the availability of information on the presence of POSI, (3) the absence of a history of neurological, neurodevelopmental, psychiatric, or learning disorders (with the exception of ADHD with hyperactivity), as well as the absence of pre-existing language disorders that could affect baseline language performance, and (4) the absence of previous chemotherapy and/or radiotherapy treatment. As the focus of the present study was on language characteristics rather than speech motor control, children were not excluded based on speech sound disorders, voice disorders, preoperative dysarthria, or having received therapy for these conditions, provided there was no evidence of an underlying language disorder.

In addition to the inclusion criteria, the patient group was further characterised by POSI status, tumour location and histology, and preoperative hydrocephalus, dysarthria and oculomotor abnormalities. POSI was reported by a clinician as present if the patient presented for at least one day postoperatively with mutism (i.e., no production of words or short sentences) or severely reduced speech (i.e., limited to single words or short sentences which can only be elicited after vigorous stimulation). Tumour location was reported after surgical resection, and could include the cerebellar vermis, the right and left hemispheres, the fourth ventricle and/or the brainstem. Tumour histology was reported as medulloblastoma, pilocytic astrocytoma, ependymoma or other. Preoperative hydrocephalus was reported as present or absent. Preoperative dysarthria and oculomotor difficulties were rated by a clinician using a five- and three-point scale, respectively, which we reclassified for the current study as absent (original score 0), or present (any higher score).

**Fig. 1:**
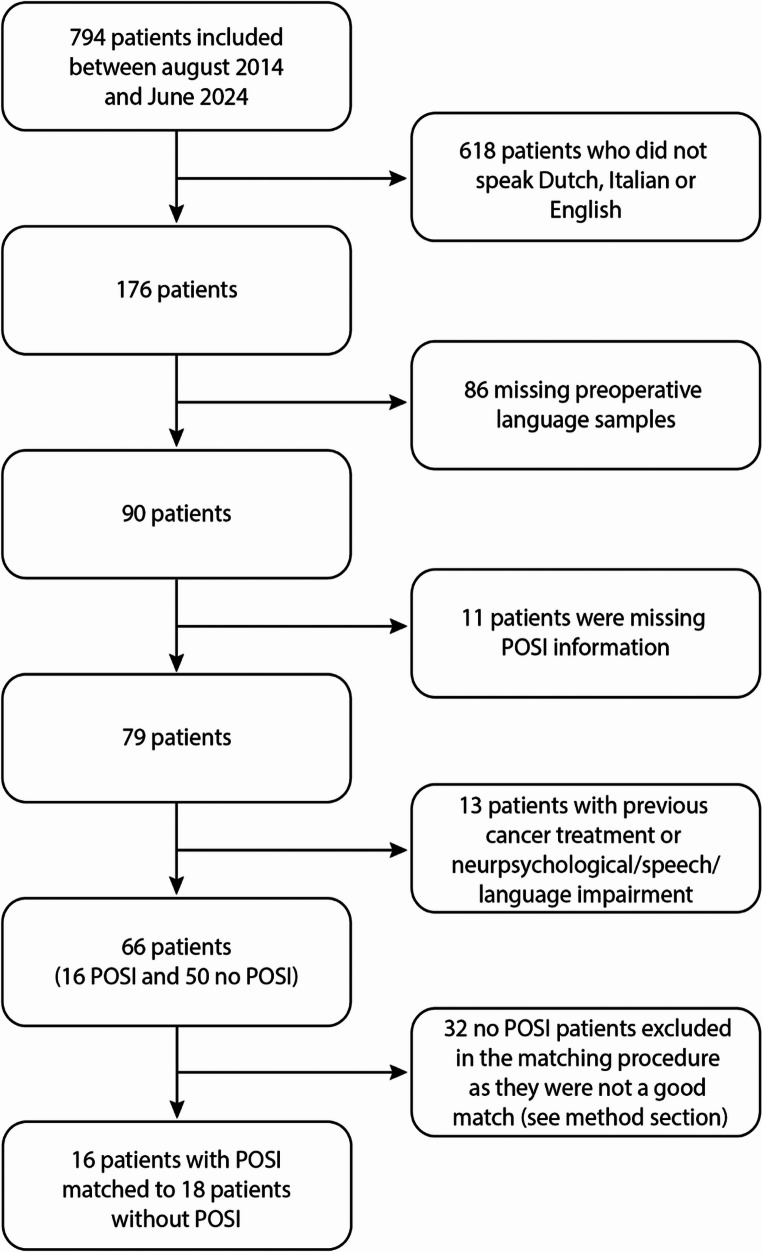
Process of participant inclusion

Participant selection is summarised in Fig. 1. Sixty-six patients met the inclusion criteria, of whom 16 developed POSI and 50 did not. Given our specific interest in language difficulties in patients with POSI, we performed an additional selection step to create a matched control group of patients without POSI. Eighteen patients who did not develop POSI were matched at the group level to the 16 patients with POSI. As the study included children speaking English and Dutch (West Germanic languages) and Italian (a Romance language), cross-linguistic structural differences (e.g., in morphological richness, pro-drop properties, and function word use) may affect measures such as MLU. Additionally, patients belonged to a wide age band, and a proportion of participants were bi- or multilingual, which may introduce variability in language performance. To minimize potential confounding effects, the groups were matched on age, sex, country and language background (i.e., mono- or multilingual), as well as presence of preoperative hydrocephalus, dysarthria, and oculomotor difficulties. Further clinical characteristics were not considered in the matching procedure to avoid creating atypical samples, given the relationship between POSI and, for instance, tumour histology and location [24].

### Materials and Procedures

The language samples were collected by a speech and language pathologist, a nurse, or a physician, typically from a few days before surgery to the day of surgery. To elicit a narrative language sample, the ERRNI – Fish Story [58] was used^1^. All clinicians administering the ERRNI received instructions from a speech and language therapist, based on the ERRNI manual. In this task, the child was presented with a wordless picture book depicting a boy visiting a pet store. Children were instructed to first look through the book silently, after which they were asked to tell the story, with the help of the pictures. The examiner was further instructed to interfere minimally but was allowed to provide encouragement to continue, such as ‘Mhm,’ or general prompts like ‘What happened next?’. However, examiners frequently intervened despite these instructions (to be explained in more detail below). The story told by the child was recorded using an audio recorder.

### Data Coding

#### Data Preparation: Global Sample Characteristics

The narrative language samples were transcribed using the transcription and annotation software ELAN [59], following a detailed protocol based on the Spontaneous Speech Analysis Procedure (STAP) [60] and the ERRNI – Fish Story [58]. Utterances that contained 20% or more unintelligible or hard-to-understand words were excluded from further analysis. Additionally, utterances in which the child mimicked the tester, general comments unrelated to the story and questions asked to the examiner were excluded from further analysis. All remaining utterances were included in the analyses described hereafter.

As mentioned above, examiners were instructed not to prompt patients but occasionally deviated from these instructions. Utterances were therefore coded into three categories: (1) *elliptic utterances*, if they were syntactically dependent on a prompt given by the tester (e.g., Examiner: *“What does the mom give to the boy?”*, Participant: *“Money”*); (2) *prompted utterances*, if they were prompted by the tester but syntactically independent (e.g., Examiner: “*What is the girl doing with the bags?”*, Participant: *“The girl is swapping the doll and the fish.“*); and (3) *free utterances*, if the child produced them spontaneously or in response to a general, neutral prompt (e.g., Examiner: “*And what do you see here?”*, Participant: “*The boy is walking to the pet store.”*). Typically, elliptic and prompted utterances are excluded from further analysis because they do not reflect the child’s independent ability to formulate a sentence and may affect language measures. However, in the current study, neither elliptic nor prompted utterances were excluded, as doing so would result in the loss of a significant portion of data.

From this pre-processing step, we calculated several global sample characteristics, which could help enhance the interpretation and contextualisation of the results. First, we calculated the sample size in *number of words* and *number of utterances*. Furthermore, the *percentage of unclear speech* was calculated, reflecting the percentage of utterances that had to be removed from the sample because they contained 20% or more hard-to-understand or unintelligible words. Finally, the *percentage of prompted utterances* was calculated to reflect the number of utterances produced with support from the examiner (either elliptic or prompted).

#### Psycholinguistic Analyses

After preparing the data, a psycholinguistic language sample analysis was performed, similar to the procedures reported by Svaldi et al. [13]. A total of 24 language measures were extracted from the samples on four levels of language processing (i.e., semantic, lexical, morphosyntactic, and phonological). See Table 1 for an overview of variables per level of language processing.

**Table 1:** Overview of language measures

Level of language processing	Standard language sample measures	Additional psycholinguistic variables
*Semantics*	-	Concreteness*
		Familiarity*
		Imageability*
		Verb instrumentality (proportion)
*Lexical*	Lexical diversity (TTR)*	Age of acquisition*
	Lexical accuracy (percentage)	Word frequency*
*Morphosyntax*	Mean length of utterance (in words)	Verb transitivity (proportion)
	Grammatical accuracy (percentage)	Unaccusativity (proportion)
	Finiteness index	Verb regularity (proportion)
*Phonology*	Phonological errors	Word length (in phonemes)*
	Cluster index	

Note. Variables marked with * were analysed separately for nouns and verbs. TTR = Type-Token Ratio. See Supplementary Material for detailed descriptions of each variable

##### **Standard Language Sample Measures**

The standard language measures at the lexical level included *Type-Token Ratio* (*TTR*) and *lexical accuracy*. TTR was calculated by dividing the number of unique nouns/verbs in the sample by the total number of nouns/verbs, including those uttered as part of hesitations, repetitions and self-corrections. TTR scores could range from 0 to 1, with scores closer to 1 expressing better performance. Lexical accuracy was determined based on whether an utterance contained lexical errors (e.g., semantic paraphasias) and was expressed as the percentage of lexically correct utterances. Morphosyntactic standard language measures included *mean length of utterance* (*MLU*), *grammatical accuracy* and *finiteness index*. MLU was calculated by dividing the total number of words in a language sample by the total number of utterances, with higher scores indicating more syntactically complex language. Grammatical accuracy was determined based on whether an utterance contained grammatical errors (e.g., errors in word order or missing elements) and was expressed as the percentage of grammatically correct utterances. The *finiteness index* was calculated by dividing the number of correctly produced inflected verbs by the total number of required inflected verbs. Scores for the finiteness index could range from 0 to 1, with scores closer to 1 expressing better performance. At the phonological level, the proportion of *phonological errors* in the sample was calculated by dividing the total number of errors by the total number of words in the sample.

##### **Additional (psycholinguistic) Variables**

For every unique noun and verb produced by the child, ratings for multiple psycholinguistic variables (e.g., imageability, frequency) were extracted, reflecting different levels of language processing. At the semantic level, *concreteness*, *familiarity*, and *imageability* ratings were extracted. On the lexical level, *AoA* and *word frequency* were considered. For the variables *verb instrumentality*,* transitivity*,* unaccusativity* and *regularity*, at the morphosyntactic level, a trained linguist determined whether the given property could be attributed to every verb produced (e.g., ‘1’ if the verb was instrumental, ‘0’ if the verb was not). Thereafter, the proportion of instrumental/transitive/regular verbs to the total number of verbs was calculated. *Unaccusativity* was calculated as the proportion of unaccusative verbs to the total number of intransitive verbs. On the phonological level, word length in phonemes was extracted from a database or, if absent, determined by a trained linguist. Additionally, the *cluster index* was calculated by dividing, per utterance, the total number of correctly produced consonant clusters by the total number of required consonant clusters. Scores could range from 0 to 1, with higher scores reflecting a more accurate production. To ensure accuracy and consistency, the aforementioned data coding was validated by the first and/or second author. See Appendix 6 for an overview of the language-specific databases used.

### Analyses

To evaluate the variables extracted from the language samples, we performed a Principal Component Analysis (PCA) [61]. Given the high number of variables included, this multivariate technique was applied to reduce the number of comparisons to be performed, by clustering variables together that contribute similarly to the variability in the data. Furthermore, considering the PCA is sensitive to the participant-variable ratio, and that we had a relatively high number of variables (24) in relation to the sample size (34), we performed a separate PCA for each level of language processing. Finally, a PCA cannot be performed when variables contain missing values. Therefore, three patients were excluded from the semantic PCA because none of their produced verbs had available concreteness or imageability ratings in the databases used.

Subsequently, the variables were evaluated for suitability for the PCA, using the Kaiser–Meyer–Olkin Measure of Sampling Adequacy (KMO) and Bartlett’s test of sphericity; variables with KMO values > 0.5^2^ and no violation of the sphericity assumption were retained. Based on the eigenvalues (> 1.0) of the components and the elbow method, we determined how many components to retain; see [61] for further explanation. Variables with a loading greater than 0.45 or less than − 0.45 were considered to contribute significantly to the variability explained by the component. All variables were then normalised and variables that had a significant contribution to the component were averaged. Where necessary, variables were recoded so that higher scores consistently reflected better performance. Those combined variables were then used for further analyses^3^.

Several variables were not eligible for inclusion in the PCA due to insufficient KMO values, indicating that these variables could not be clustered in components. Those variables were therefore compared between groups separately. Extracted components from the PCA and separate variables were compared between groups separately using linear models [62], including *group* (POSI vs. no POSI, i.e., habitual speech) as a predictor and *age* and *country* as covariates. Additionally, we added *group* × *age* interactions to the models, given the higher risk of developing POSI for younger children. For components including TTR nouns and/or TTR verbs, we added *number of words* as a fixed effect to the model, given TTR’s known sensitivity to sample size [46]. Additionally, we ran linear models for the global sample characteristics (i.e., sample size in words and utterances, intelligibility and prompting). All statistical analyses were performed using RStudio [63].

## Results

### Patient Sample

Table 2 presents the demographic characteristics of the final patient groups. Across the total sample, participants ranged in age from 3;5 to 16;0 years (median = 8;8, IQR = 6;5–12;3; M = 9;0, SD = 3;5). One child in the POSI group had previously received speech support for a voice disorder. In the no-POSI group, one patient had a diagnosis of ADHD with hyperactivity, and one patient had documented unilateral preoperative damage to the right VIIIth cranial nerve, which was considered a proxy indicator of hearing status in the absence of systematic hearing data. The groups did not differ in tumour histology and tumour location, although a trend towards a group difference in tumour location was observed. See Appendix Table 5 for individual demographic and clinical information.

**Table 2:** Demographic and clinical background information per group

	POSI (*n* = 16)	no POSI (*n* = 18)	χ^2a^/t	*p*
Age (Y; M)			*t = -*0.214	0.832
Range	3;9–14;2	3;5–16;0		
* M* (*SD*)	8;10 (3;3)	9;1 (3;8)		
Median (IQR)	8;7 (6;3–12;3)	8;6 (6;5–12;1)		
Sex, *n* (%^b^)			χ^2^ = 0.007	1.000
Male	10 (63)	11 (61)		
Female	6 (37)	7 (39)		
Country, *n* (%)			χ^2^ = 0.092	1.000
Italy	7 (44)	8 (44)		
The Netherlands	3 (19)	4 (22)		
United Kingdom	6 (38)	6 (33)		
Language background, *n* (%)			χ^2^ = 0.993	1.000
Monolingual	13 (81)	14 (78)		
Bi- and multilingual	3 (19)	3 (17)		
Unknown	0 (0)	1 (6)		
Tumour location, *n* (%)			χ^2^ = 8.543	0.065
Left cerebellar hemisphere	1 (6)	1 (6)		
Right cerebellar hemisphere	0 (0)	6 (38)		
Vermis	5 (28)	7 (44)		
Fourth ventricle	13 (72)	7 (44)		
Brainstem	4 (22)	7 (44)		
Tumour histology, *n* (%)			χ^2^ = 6.359	0.156
Medulloblastoma	10 (63)	6 (33)		
Pilocytic astrocytoma	2 (13)	9 (50)		
Ependymoma	1 (6)	1 (6)		
Other	2 (13)	2 (11)		
Unknown	1 (6)	0 (0)		
Pre-op dysarthria, *n* (%)			χ^2^ = 0.694	0.834
Present	2 (13)	1 (6)		
Absent	13 (81)	15 (83)		
Unknown	1 (6)	2 (11)		
Pre-op hydrocephalus, *n* (%)			χ^2^ = 0.034	1.000
Present	12 (75)	13 (72)		
Absent	4 (25)	5 (28)		
Pre-op oculomotor difficulties, *n* (%)			χ^2^ = 0.174	1.000
Present	9 (56)	9 (50)		
Absent	5 (31)	6 (33)		
Unknown	2 (13)	3 (17)		

Note. POSI = Postoperative Speech Impairment, defined as mutism or severely reduced speech; No POSI = habitual speech; Y;M = age in years and months; Other = Atypical Teratoid/Rhabdoid Tumour and other tumour types; Pre-op = preoperative.

^a^Chi square test was performed using Monte Carlo simulation (10,000 replicates) to account for small cell counts and limited sample size.

^b^Percentages may not total exactly 100% due to rounding

### Global Sample Characteristics

In Table 3, the results of the analysis of the global sample characteristics are reported. The percentage of unclear speech that had to be excluded from further analyses was significantly higher in the group who later developed POSI compared to the group with habitual speech (β = -14.455, *p* = .024). We also found a significant interaction between *group* and *age*. In the POSI group, intelligibility was lower in younger children but improved with increasing age, whereas in children with habitual speech, intelligibility was relatively similar across ages (β = 0.152, *p* = .007). Additionally, a near-significant difference was observed in the proportion of utterances produced with examiner support (either prompted or elliptic; β = 48.754, *p* = .064), indicating a tendency for children who later developed POSI to receive more prompts from the examiner. No main effect of *group* was found in the number of words or utterances that the child produced when telling the story. *Age* did not show a significant main effect on any of the global sample characteristics. *Country* significantly impacted the percentage of unclear speech and the sample size in number of utterances. See Appendix Table 8 for the results of the complete models including predictors and covariates.

**Table 3:** Linear models for global sample characteristics

	POSI	No POSI	Group β (SE, *p*)	Age × group β (SE, *p*)	*R*²
#Words	105	132	-42.75 (55.20, 0.445)	0.016 (0.48, 0.739)	0.18
#Utterances	18.8	19.9	2.91 (5.57, 0.605)	-0.03 (0.05, 0.484)	0.17
%Unclear	3.9%	1.9%	-14.46 (6.06, 0.024)	0.15 (0.05, 0.007)	0.22
%Prompted	23.6%	11.6%	48.75 (25.31, 0.064)	-0.35 (0.22, 0.127)	0.23

Note. POSI = postoperative speech impairment, defined as mutism or severely reduced speech; No POSI = habitual speech; #Words = sample size in number of words; #Utterances = sample size in number of utterances; %Unclear = percentage of hard-to-understand and unintelligible speech; SE = standard error

### Psycholinguistic Analysis

#### Principal Component Analysis

In the Principal Component Analysis, two components (C1 and C2) were extracted per level of language processing. At the semantic level, all variables were included in the PCA. For Semantics C1, the variables with a significant loading were *concreteness verbs*,* imageability nouns*,* imageability verbs*, and *instrumentality verbs*, and for Semantics C2 these were *concreteness nouns* and *familiarity nouns*. At the lexical level, the variables *AoA verbs* and *frequency nouns* were not suitable for inclusion in the PCA due to insufficient KMO values. The components extracted from the remaining variables were C1, where *lexical correctness*,* AoA nouns* and *frequency verbs* had a significant contribution, and C2, containing the variables *TTR verbs* and *TTR nouns*. At the morphosyntactic level, *unaccusativity* was excluded from further analysis, as only 6 children produced unaccusative verbs. *Regularity* was not included in the PCA due to an insufficient KMO value. Two components were extracted from the remaining variables: C1 containing *grammatical correctness* and *finiteness index*, and C2 containing *MLU* and *transitivity*. At the phonological level, all variables were included in the PCA. In C1, *word length verbs* and *word length nouns* had a significant loading. In C2, the variables with a significant loading were *phonological errors* and *cluster index*. See Appendix Table 7 for the full overview of the variables and their loadings in the components.

#### Group Comparisons

Linear models were constructed for each component and for the separate variables not included in the PCA, including *number of words* (C2), *age* and *country* as covariates, *group* as a predictor, and the interaction *group* × *age*. See Table 4 for an overview of the models for each component and separate variable and see Appendix Table 8 for the results of the complete models including predictors and covariates.

**Table 4:** Linear model per component or separate variable

Component/variable	Group β (SE, *p*)	Age × group β (SE, *p*)	*R*²
Semantic			
C1: Concr. V+Imageab. V+	-0.19 (0.12, 0.103)	-0.00 (0.00, 0.666)	0.54
Instrum. V+Imageab. N			
C2: Concr. N + Fam. N	-0.30 (0.57, 0.605)	0.00 (0.00, 0.706)	0.65
Lexical			
C1: Lex. cor + AoA N+	-0.73 (0.41, 0.086)	0.01 (0.00, 0.137)	0.77
Freq. V			
C2: TTR N + TTR V	0.51 (0.77, 0.517)	-0.00 (0.01, 0.722)	0.31
AoA V	-0.22 (0.64, 0.739)	0.00 (0.01, 0.535)	0.63
Freq. N	1.25 (1.02, 0.232)	-0.01 (0.01, 0.143)	0.07
Morphosyntax			
C1: Gram. cor.+Finite. ind.	-0.96 (0.84, 0.261)	0.01 (0.01, 0.307)	0.06
C2: MLU+Trans. V	-1.23 (0.95, 0.206)	0.01 (0.01, 0.162)	0.15
Regularity V	-0.08 (0.74, 0.916)	0.00 (0.01, 0.994)	0.52
Phonology			
C1: Length V+Length N	0.05 (0.22, 0.810)	-0.00 (0.00, 0.895)	0.95
C2: Phon. err.+Clust. ind.	-0.05 (0.04, 0.257)	-0.00 (0.76, 0.451)	0.17

Note. C = Component; N = Nouns; V = Verbs; Concr. = Concreteness; Imageab. = Imageability; Instrum. = Instrumentality; Fam. = Familiarity; Lex. cor. = Lexical correctness; AoA = Age of acquisition; Freq. = Frequency; TTR = Type-Token Ratio; Gram. cor. = grammatical correctness; Finite. ind. = finiteness index; Trans. = Transitivity; Phon. err. = Phonological errors; Clust. ind. = Cluster index; SE = standard error

*Country* had a significant effect on the components/variables Semantic C1, Semantic C2, Lexical C1, AoA verbs, verb regularity and Phonology C1 and C2. Additionally, *age* significantly impacted Frequency nouns, and *number of words* had a significant effect on the component Lexical C2, including TTR nouns and verbs. *Group* appeared not to be a significant predictor of the scores for any of the components or separate variables. No significant *age* × *group* interactions were found for any of the components or separate variables.

## Discussion

To examine whether preoperative language characteristics are related to the emergence of POSI, we compared preoperative narrative language samples of 16 patients who developed POSI and those of 18 patients who did not following neurosurgical resection for a PFT. Younger children with POSI produced more unclear speech relative to older children with POSI; this effect was not seen for children without POSI. In contrast, at the level of language, the psycholinguistic analyses showed no differences between the two groups in the measures reflecting semantic, lexical, morphosyntactic, and phonological processing, and no interactions between group and age. In this section, we will discuss the group comparisons and how this relates to previous research on this population.

### Global Sample Characteristics

Considering the global sample characteristics, the percentage of unclear speech that had to be excluded from further analysis was higher in the patients who later developed POSI compared to those who did not. Bianchi et al. [14] characterised preoperative impairments in the vast majority of patients who went on to develop mutism as a *phonological disorder*, implying a disorder at the level of language. However, they also referred to these difficulties as a *phonetic disorder* and *apraxia of speech*, which are motor speech disorders, leaving some uncertainty about the exact nature of these difficulties. While unclear speech was excluded from further analysis, motor speech-related factors potentially contributed to the speech being unintelligible, such as respiration, phonation, resonance, prosody, articulation, and speech rate [64, 65]. Several studies reported preoperative difficulties in the domain of speech in children who developed mutism, such as dysarthria [15, 65], ataxia [65–67] and apraxia of speech [15], although these did not extensively assess the nature of these speech difficulties. A more systematic, in-depth analysis of preoperative speech, intelligibility and speech sound errors could provide more insight into the phonological and/or speech impairments these patients experience and whether these could be a risk factor for the development of POSI.

Interestingly, age differentially affected the percentage of unclear speech across groups. In children who later developed POSI, unclear speech was more frequent in younger children and decreased with increasing age, whereas no age-related association was observed in children who did not develop POSI. This aligns with the reported higher risk of speech impairments in younger children, namely for the occurrence of mutism or reduced speech [5, 6]. Mutism or reduced speech is hypothesised to be a form of cerebello-cerebral diaschisis, characterised by damage to the connections between the cerebellum and cerebrum, resulting in hypoactivity of the cerebral hemispheres [68, 69]. These connections may be more vulnerable to damage in children, due to the incomplete maturation of the brain, resulting in a higher incidence of mutism or reduced speech [27]. The presence of this age effect already before neurosurgical resection suggests that not only the surgical intervention [24], but also the tumour itself may have a stronger impact on these pathways in younger children.

Additionally, we observed an imbalance in the amount of prompting provided by the testers between groups, with more prompting for the POSI group, albeit just above the significance threshold. This occurred despite instructions to interfere minimally and only provide general prompts when the child needed encouragement [58]. This pattern might suggest that the adynamic verbal output pattern often observed after neurosurgical resection [18] could already be present to some extent in the preoperative stage, prompting testers to provide more support. However, this prompting should be investigated more systematically, in greater detail and in a larger group of patients before firm conclusions can be drawn.

### Psycholinguistic Analysis

At the level of language, none of the measures in our psycholinguistic analysis differentiated children who developed POSI from those who did not based on their preoperative abilities. These results are in contrast with previous research by Di Rocco et al. [15], who found a lower MLU and problems with lexical naming and verbal fluency for children who developed mutism before surgery, and Bianchi et al. [14], who expanded Di Rocco et al.’s sample, suggested a relationship between preoperative language performance and mutism. Critically, MLU was defined by Di Rocco et al. [15] based on parental report [70, 71]. This measure may not be entirely comparable to the typical MLU calculation in linguistic studies, which reflects the length of syntactic units and distinctions between devices such as conjunction vs. subordination, which would be difficult for parents to judge [72]. Furthermore, motor speech symptoms (rather than language difficulties), such as impaired coordination of respiration, phonation, and articulation, may lead to more frequent pauses, potentially causing sentences to be judged by parents as shorter. In line with this, a perceptual analysis of speech carried out in the European CMS Study [73] by our team showed that children who went on to develop POSI speak in shorter phrases before surgery, compared to those who do not. Similarly, performance on language tasks such as picture naming or verbal fluency may also be influenced by poor motor speech, especially when the tasks are timed (as is the case of verbal fluency) or reaction times are taken into account, as done in some picture naming tests [74, 75].

Additionally, performance on language tasks is not solely determined by language or speech abilities. Verbal fluency, for instance, also relies on neuropsychological processes such as executive functioning and processing speed [76, 77]. The group differences in verbal fluency found by Di Rocco and colleagues could therefore also be driven by difficulties in these domains. Horne et al. [78] and Cámara et al. [19] reported postoperative impairments in executive functioning and processing speed. Given the overall poorer preoperative neuropsychological status of patients who developed mutism reported by Mariën [68], these difficulties are likely at least to some extent already present before neurosurgical resection.

Combined, this suggests that children with and without mutism or reduced speech may differ in their (motor) speech or overall neuropsychological status before neurosurgical resection, which may affect their performance on language tasks, while differences in language itself may be absent. From a clinical perspective, the present findings therefore do not support the inclusion of preoperative language measures in risk prediction models for postoperative mutism or reduced speech. Instead, the observed preoperative differences in speech intelligibility suggest that speech-related measures may be of interest as potential clinical markers for POSI in future risk prediction models, although this requires further investigation. Nevertheless, children with PFTs in general may still exhibit preoperative language impairments (relative to healthy children, not examined in this study) due to tumour presence and growth. This was shown for word finding by Persson et al. [29]. Further studies could provide more insight into the extent of difficulty and the specific levels of language affected.

Something that has to be taken into account when interpreting the results is the type of tumour diagnosed in patients. The groups were not matched on tumour type, as this would create atypical groups, given the relatedness of tumour type (namely medulloblastomas) to emergence of mutism or reduced speech [4, 24]. In line with this, the proportion of medulloblastomas is relatively (albeit non-significantly) higher in our POSI group (63%), compared to the no POSI group (33%). However, Persson et al. [29] did not find a relation between tumour type and word-finding difficulties. They hypothesize that, for medulloblastomas, mutism or reduced speech may not be related to the tumour itself, but to the high-risk surgery medulloblastomas require. This could also explain the present findings, where a higher proportion of medulloblastomas was found in the group that later developed POSI, but no group differences in language were found before neurosurgical resection; perhaps postoperative language differences found in earlier studies are related to more aggressive surgery in the POSI group, driven by a higher proportion of medulloblastomas. Still, we find more unclear speech in patients with POSI before surgery, indicating that not all impairments can be explained this way. Further research on the relationship between tumour type, location, and preoperative speech and language performance is needed to better understand the impact of tumour characteristics on performance.

### Limitations and Suggestions for Future Research

A limitation of this study concerns the type of test (i.e., a picture-based narrative task) that was used to collect a language sample, which might have affected the quality of the language samples extracted. Compared to storytelling tasks, story retelling tasks have been associated with better narrative macrostructure [79], and picture-sequence narrative tasks with greater lexical diversity than single-picture tasks [80]. Furthermore, in a picture-based narrative task, the predetermined content may have limited children in using their linguistic abilities to their full potential and restricted the potential range of psycholinguistic variable values, such as imageability and word length. Compared to no-visual narrative tasks, samples elicited with a picture-based task were found to be less elaborate [80], less lexically complex [81], and had shorter MLUs [80]. Moreover, a free no-visual conversation task was found to better capture atypical patterns in semantic properties such as concreteness and imageability and to generally detect more atypical language profiles than a picture-description task [13]. Although we believe differences between groups might primarily be in the domain of speech, the task used might have made it difficult to capture subtle language differences. Opting for more naturalistic approaches (e.g., parent-child or examiner-child interactions; see, for instance, Ellis Weismer et al. [82]) may provide a more ecologically valid and comprehensive measurement of language abilities. Nonetheless, it should be noted that the European CMS study is a large-scale study across many centres and languages. The ERRNI procedure [58], using a picture-book-based narrative, creates a setting where language samples can be gathered in a standardised way, despite the large variability in settings, languages and professional backgrounds of those administering the task. Although some variability related to these factors cannot be entirely ruled out, other, more naturalistic or interview-based data collection procedures might introduce much more variability and require even greater expertise from examiners, which may have a detrimental effect on the feasibility of the study or the quality of the data.

Furthermore, the way patients were classified as experiencing mutism or reduced speech may have impacted our results. In the European CMS study, decisions about whether a child showed mutism or reduced speech were not made by speech and language therapists, but by clinicians such as surgeons, paediatricians or nursing staff. While agreement on identifying mutism as a complete absence of speech is likely high, the categorisation of reduced speech may be more variable. What one rater considers a clinically significant reduction in speech may not be judged as such by another. This heterogeneity may have introduced noise into the POSI/no POSI classification, potentially reducing our ability to detect associations with preoperative language abilities. Future research may benefit from standardised criteria or rater training, or from involving speech and language therapists in the diagnostic process, to improve the reliability of POSI classification.

Another limitation concerns the availability of further language and neuropsychological assessment data. Because we performed a language sample analysis, 86 patients for whom there was no sample available had to be excluded. Information on the reason for not performing the language task was often not available, but this could be related to the characteristics or neuropsychological status of the patients. Mariën et al. [68] proposed a relationship between neuropsychological status and the emergence of mutism, making the excluded group particularly interesting for future research. Although language test administration is difficult in this group, future research could focus on suitable tests to gain a better understanding of the language abilities of children with worse neuropsychological status. In addition, no standardised neuropsychological or formal language test scores were available in the present dataset. The inclusion of such measures may provide additional context for interpreting language findings and could contribute to a more comprehensive understanding of the broader cognitive profile associated with POSI in future studies.

## Conclusion

This study aimed to identify risk factors in the domain of language that could be related to the emergence of POSI, using a comprehensive analysis of preoperative language samples. The global sample characteristics and the linguistic abilities of patients who developed POSI were compared to those of patients who did not develop POSI. Results revealed a higher proportion of unclear speech in the group that later developed POSI. The linguistic analysis of the language samples did not reveal any group differences. Our results indicate that preoperative differences between the two groups may be primarily related to motor speech rather than to the microstructural aspects of language assessed through our psycholinguistic analyses. The language differences between patients with and without POSI observed postoperatively may thus be correlated with the effects of neurosurgical tumour resection.

This study adds to the limited body of preoperative research performed in this population and suggests that already at the preoperative stage, there might be speech characteristics that are related to the emergence of postoperative mutism or reduced speech. Additional research is needed to further explore the predictive value of speech characteristics (which we predict will show more prominent group differences, given our findings on intelligibility). Such advances will help form an increasingly accurate risk prediction of the development of mutism or reduced speech.

## Supplementary Information

Below is the link to the electronic supplementary material.

Supplementary Material 1

## Figures and Tables

**Table 5:**
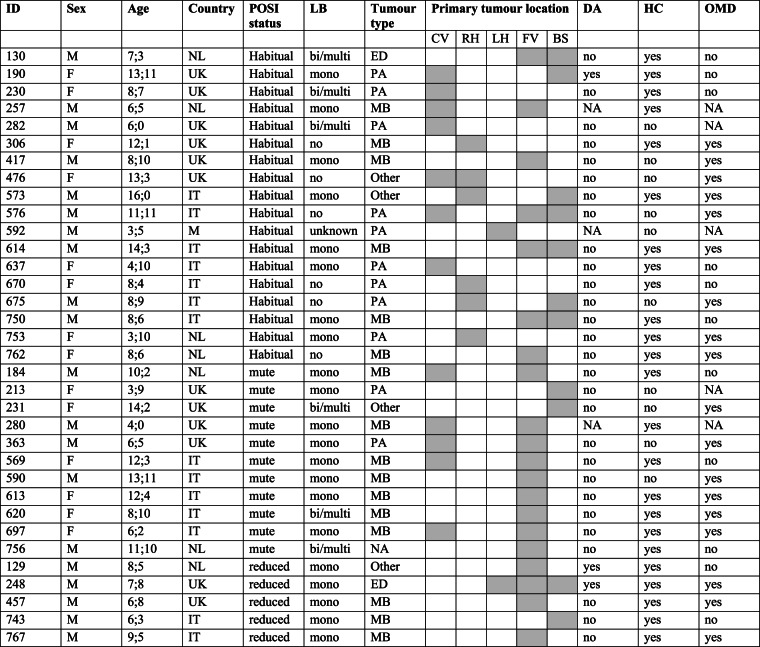
Individual demographic and clinical information

Note. ID = participant ID; F = female; M = male; Age = age in years and months; Country = country where patients were treated (NL = Netherlands; UK = United Kingdom; IT = Italy); LB = language background (mono = monolingual; bi-/multi = bi- or multilingual); POSI status = postoperative speech impairment (Habitual = habitual speech; reduced = reduced speech; mute = mutism); MB = medulloblastoma; PA = pilocytic astrocytoma; ED = ependymoma; Other = Atypical Teratoid/Rhabdoid Tumour and other tumour types; VM = vermis; LH = left hemisphere; RH = right hemisphere; FV = fourth ventricle; BS = brain stem; DA = dysarthria; HC = hydrocephalus; OCM = oculomotor difficulties.

## Data Availability

The paper reports a secondary analysis of data from the European CMS study. Requests for access to and reuse of the data should be directed to the principal investigator of the European CMS study, René Mathiasen (Rene.Mathiasen@regionh.dk).

## Appendix

**Table 6:** Overview of databases used for extracting psycholinguistic values

Property	English	Dutch	Italian
Concreteness	[42]	[42]	Montefinese et al., (2014)
Imageability	[41]	Van Loon-Vervoorn, (1984)	Montefinese et al., (2014)
Familiarity	[41]	Hermans & De (Houwer, 1994)	Montefinese et al., (2014)
Age of acquisition	[48]	[42]	ItAoA, Montefinesse et al., (2019)
Frequency	van Heuven et al., (2014)	SUBTLEX-NL (Keuleers et al., 2010)	Montefinesse et al., (2014)
Word length	CLEARPOND (Marian et al., 2012)	DutchPOND (Marian et al., 2012)	Self constructed

**Table 7:** Components and variable loadings extracted from the PCA

Level of Language Processing	Variable	C1	C2
Semantics	Concreteness verbs	**0.92**	0.16
	Concreteness nouns	0.21	**0.87**
	Imageability verbs	**0.95**	-0.11
	Imageability nouns	**0.66**	0.14
	Familiarity nouns	0.02	**0.90**
	Instrumentality verbs	**-0.78**	-0.19
Lexical	TTR nouns	0.26	**0.80**
	TTR verbs	0.02	**0.90**
	Lexical correctness	**-0.79**	-0.23
	AoA nouns	**0.74**	0.18
	Frequency verbs	**0.84**	-0.01
Morphosyntax	MLU	0.42	**0.60**
	Grammatical correctness	**0.96**	0.12
	Finiteness index	**0.92**	0.30
	Transitivity	0.08	**0.92**
Phonology	Word Length verbs	**0.95**	-0.03
	Word Length nouns	**0.95**	0.07
	Phonological errors	-0.04	**0.95**
	Cluster Index	-0.11	**-0.94**

Note. Values in **bold** relate to the variables that are normalised and averaged to construct the component; C1 = Component 1; C2 = Component 2

**Table 8:** Complete linear models including predictors and covariates

Number of words					Number of utterances				
**Predictor**	**β**	**SE**	**t**	***p***	**Predictor**	**β**	**SE**	**t**	***p***
(Intercept)	105.61	37.23	2.84	0.008	(Intercept)	19.62	3.75	5.23	< 0.001
Group	-42.75	55.19	-0.77	0.445	Group	2.91	5.57	0.52	0.605
Age	0.41	0.30	1.37	0.182	Age	0.03	0.03	1.02	0.316
Country (NL)	-36.61	24.64	-1.49	0.148	Country (NL)	-4.64	2.48	-1.87	0.073
Country (UK)	-31.09	21.09	-1.47	0.152	Country (UK)	-6.45	2.13	-3.03	0.005
Group × Age	0.16	0.48	0.34	0.739	Group × Age	-0.03	0.05	-0.71	0.484
**Model fit**: R² = 0.30; Adj. R² = 0.18; F(5, 28) = 2.43; *p* = .060; Residual SE = 52.09					**Model fit**: R² = 0.30; Adj. R² = 0.17; F(5, 28) = 2.39; *p* = .063; Residual SE = 5.25				
% unclear speech					% prompted utterances				
**Predictor**	**β**	**SE**	**t**	***p***	**Predictor**	**β**	**SE**	**t**	***p***
(Intercept)	6.44	4.09	1.58	0.126	(Intercept)	35.67	17.07	2.09	0.046
Group	-14.46	6.06	-2.39	0.024	Group	48.75	25.31	1.93	0.064
Age	-0.05	0.03	-1.57	0.127	Age	-0.17	0.14	-1.22	0.233
Country (NL)	-3.18	2.71	-1.18	0.249	Country (NL)	-4.43	11.30	-0.39	0.698
Country (UK)	4.83	2.32	2.08	0.046	Country (UK)	-13.88	9.67	-1.44	0.162
Group × Age	0.15	0.05	2.89	0.007	Group × Age	-0.35	0.22	-1.57	0.127
**Model fit**: R² = 0.33; Adj. R² = 0.21; F(5, 28) = 2.71; *p* = .040; Residual SE = 5.72					**Model fit**: R² = 0.34; Adj. R² = 0.22; F(5, 28) = 2.92; *p* = .031; Residual SE = 23.89				
Semantics C1					Semantics C2				
**Predictor**	**β**	**SE**	**t**	***p***	**Predictor**	**β**	**SE**	**t**	***p***
(Intercept)	0.55	0.24	2.28	0.031	(Intercept)	0.69	0.38	1.81	0.082
Group	-0.05	0.36	-0.13	0.900	Group	-0.30	0.56	-0.52	0.605
Age	-0.00	0.00	-0.43	0.672	Age	-0.00	0.00	-0.65	0.524
Country (NL)	-0.36	0.16	-2.22	0.035	Country (NL)	-1.91	0.25	-7.57	< 0.001
Country (UK)	-0.86	0.14	-6.25	< 0.001	Country (UK)	-0.10	0.22	-0.46	0.650
Group × Age	-0.00	0.00	-0.44	0.666	Group × Age	0.00	0.00	0.38	0.706
**Model fit**: R² = 0.61; Adj. R² = 0.54; F(5, 28) = 8.82; *p* < .001; Residual SE = 0.34					**Model fit**: R² = 0.70; Adj. R² = 0.65; F(5, 28) = 13.07; *p* < .001; Residual SE = 0.53				
Lexical C1					Lexical C2				
**Predictor**	**β**	**SE**	**t**	***p***	**Predictor**	**β**	**SE**	**t**	***p***
(Intercept)	-0.76	0.28	-2.74	0.011	(Intercept)	-0.38	0.59	-0.65	0.524
Group	-0.73	0.41	-1.78	0.086	#Words	-0.01	0.00	-2.63	0.014
Age	0.00	0.00	0.13	0.898	Group	0.51	0.77	0.66	0.517
Country (NL)	1.19	0.18	6.47	< 0.001	Age	0.01	0.00	1.63	0.115
Country (UK)	1.58	0.16	9.99	< 0.001	Country (NL)	0.56	0.35	1.59	0.123
Group × Age	0.01	0.00	1.53	0.137	Country (UK)	0.59	0.30	1.96	0.061
**Model fit**: R² = 0.80; Adj. R² = 0.77; F(5, 28) = 22.55; *p* < .001; Residual SE = 0.39					**Model fit**: R² = 0.44; Adj. R² = 0.31; F(6, 27) = 3.48; *p* = .011; Residual SE = 0.72				
Age of Acquisition verbs					Frequency nouns				
**Predictor**	**β**	**SE**	**t**	***p***	**Predictor**	**β**	**SE**	**t**	***p***
(Intercept)	-0.52	0.43	-1.21	0.237	(Intercept)	-1.20	0.69	-1.74	0.093
Group	-0.22	0.64	-0.34	0.739	Group	1.25	1.02	1.22	0.232
Age	0.00	0.00	0.58	0.565	Age	0.01	0.01	2.35	0.026
Country (NL)	1.76	0.29	6.12	< 0.001	Country (NL)	-0.26	0.46	-0.56	0.578
Country (UK)	-0.38	0.25	-1.54	0.135	Country (UK)	-0.23	0.39	-0.59	0.561
Group × Age	0.00	0.01	0.63	0.535	Group × Age	-0.01	0.01	-1.51	0.143
**Model fit**: R² = 0.69; Adj. R² = 0.63; F(5, 28) = 12.38; *p* < .001; Residual SE = 0.61					**Model fit**: R² = 0.21; Adj. R² = 0.07; F(5, 28) = 1.48; *p* = .228; Residual SE = 0.97				
Morphosyntax C1					Morphosyntax C2				
**Predictor**	**β**	**SE**	**t**	***p***	**Predictor**	**β**	**SE**	**t**	***p***
(Intercept)	-0.20	0.56	-0.35	0.729	(Intercept)	-0.21	0.64	-0.33	0.743
Group	-0.96	0.84	-1.15	0.261	Group	-1.23	0.95	-1.29	0.206
Age	0.00	0.00	0.66	0.514	Age	0.00	0.01	0.74	0.467
Country (NL)	-0.47	0.37	-1.25	0.221	Country (NL)	-0.57	0.42	-1.34	0.191
Country (UK)	0.11	0.32	0.35	0.732	Country (UK)	-0.29	0.36	-0.79	0.439
Group × Age	0.01	0.01	1.04	0.307	Group × Age	0.01	0.01	1.44	0.162
**Model fit**: R² = 0.20; Adj. R² = 0.06; F(5, 28) = 1.40; *p* = .255; Residual SE = 0.79					**Model fit**: R² = 0.28; Adj. R² = 0.15; F(5, 28) = 2.15; *p* = .089; Residual SE = 0.90				
Verb regularity					Phonology C1				
**Predictor**	**β**	**SE**	**t**	***p***	**Predictor**	**β**	**SE**	**t**	***p***
(Intercept)	0.86	0.50	1.73	0.095	(Intercept)	0.67	0.15	4.40	< 0.001
Group	-0.08	0.74	-0.11	0.916	Group	0.05	0.22	0.24	0.810
Age	0.00	0.00	0.03	0.978	Age	0.00	0.00	1.89	0.069
Country (NL)	-1.77	0.33	-5.40	< 0.001	Country (NL)	-1.01	0.10	-10.09	< 0.001
Country (UK)	-1.32	0.28	-4.69	< 0.001	Country (UK)	-2.04	0.09	-23.81	< 0.001
Group × Age	0.00	0.01	-0.01	0.994	Group × Age	0.00	0.00	-0.13	0.895
**Model fit**: R² = 0.59; Adj. R² = 0.52; F(5, 28) = 8.09; *p* < .001; Residual SE = 0.69					**Model fit**: R² = 0.96; Adj. R² = 0.95; F(5, 28) = 128.00; *p* < .001; Residual SE = 0.21				
Phonology C2									
**Predictor**	**β**	**SE**	**t**	***p***					
(Intercept)	0.45	0.03	16.83	< 0.001					
Group	-0.05	0.04	-1.16	0.258					
Age	0.00	0.00	1.12	0.272					
Country (NL)	0.04	0.02	2.11	0.044					
Country (UK)	0.03	0.02	1.76	0.090					
Group × Age	0.00	0.00	0.76	0.451					
**Model fit**: R² = 0.30; Adj. R² = 0.17; F(5, 28) = 2.35; *p* = .067; Residual SE = 0.04									

## Footnotes

1 Although the ERRNI provides normative data from 4 years of age and has been standardised in English, the present study used the picture book only as a narrative elicitation tool. It served to generate data which were used for group comparison; no normative scores were calculated.

2 Given that the main goal of performing the PCA was dimensionality reduction, we choose to be lenient with this cut-off. Therefore, if there were any variables that had a slightly lower (e.g., 0.48) KMO value, but did cluster together with other variables in a meaningful way later on in the analyses, we decided to include those variables in the analyses.

3 In a PCA, every variable has a loading onto every component, some stronger than others (expressed by the loading value). It is possible to extract components that incorporate all variables while considering their specific loadings. However, this leads to less interpretable data, as each component becomes a mixture of all variables. Therefore, we choose to use the PCA as a guide to identify which variables can be clustered together and we averaged the scores of only those variables that made a significant contribution to the component.
