# Supplementary material for: Analysis of Presurgical Language in Children with Posterior Fossa Tumours Relative to Postoperative Speech Outcomes: Findings from the European CMS Study
Source: Cerebellum. 2026 Apr 10;25(2):48. doi: 10.1007/s12311-026-01987-3 (PMC13068704; doi:10.1007/s12311-026-01987-3)
Supplement: Supplementary file 1 — Supplementary Material 1 [file 12311_2026_1987_MOESM1_ESM.docx]

**Supplementary Material**

for

**Analysis of pre-surgical language in children with posterior fossa tumours relative to postoperative speech outcomes: findings from the European CMS study**

In this supplementary material, we provide an extended overview of the psycholinguistic properties included in our analyses. This overview elaborates on how these properties relate to different levels of language processing and how they have been used in previous experimental research in several clinical populations, such as in children with language disorders [1–8] and people with aphasia [9–21]. Additionally, psycholinguistic properties have been used to reveal level-specific language impairments in a wide variety of tasks, including (semi-)spontaneous language [4,20,22–24]. In what follows, we provide a more detailed description of four levels of language processing involved in production (i.e., semantics, lexical, morphosyntax, and phonology). This description is based on the language generation model proposed by Levelt [25]. For each level we outline which variables can be extracted from a language sample and explain how these measures may serve as markers of impairment.

***Semantics***

Semantics relates to the meaning of words. At this level, speakers construct a conceptual representation of what they want to express [25]. The ease of retrieving semantic information needed to convey meaning can be influenced by semantic complexity. There are several psycholinguistic properties that reflect the semantic complexity of concepts.

*Concreteness* is a property reflecting how perceptible or tangible a word is [26]. ‘Capability’, for instance, has a lower concreteness rating than ‘tree’. Highly concrete words contain richer sensory and perceptual features, which means they carry more semantic information. Highly concrete words, associated with richer and more accessible contextual information [27], have been reported to have a facilitating effect on word recognition and production in children with a developmental language disorder (DLD) [8], children treated for a PFT [24], neurotypical adults [28], and people with aphasia [29], with highly concrete words being easier to recognise/produce. A reverse effect of concreteness was found in people with semantic dementia, who seem to have more difficulties with concrete words [30,31]. *Imageability* is closely related to concreteness and expresses how easily a word evokes a mental image [32]. Although concreteness and imageability are correlated (e.g., ‘capability’ and ‘tree’ have similar ratings on both properties), they are distinct. For instance, highly imageable words may be less concrete, such as emotion words like ‘panic’ [33]. Highly imageable words seem to be easier and faster to name in people with aphasia [29] and children with a language disorder [8]. Additionally, an increased use of highly imageable and concrete words in the spontaneous language of children treated for a PFT was found [24]. Furthermore, imageability has been linked to expressive vocabulary size: children with a smaller vocabulary size produced words of a higher imageability, indicating a heavier reliance on perceptual-based language production, while children with larger vocabularies produced words of a lower imageability [34].

*Familiarity* reflects the extent to which a word feels known or recognizable to someone, based on past experiences or exposure. More familiar items were easier to access and name for people with aphasia [15,21] and to name and  generate in children with a language disorder [1,8].

Finally, *verb instrumentality* expresses whether an action is performed with an instrument (not a body part), such as ‘cutting’ for which the instrument ‘scissors’ is needed as opposed to ‘walking’. The instrument adds semantic complexity, but there is no consensus on the effect of instrumentality on language performance. It has been reported to have a facilitating effect in adults with aphasia [16] and a hindering [7], or no effect [35] in children with language disorders.

Although the opposite has also been shown [7,36], the vast majority of studies suggest that increased semantic complexity (i.e., more semantic information) has a facilitating effect on language processing. A heavier reliance on semantically complex words (e.g., more concrete, imageable and familiar nouns/verbs and more instrumental verbs), reflecting the need for facilitatory semantic information, could therefore indicate semantic impairments.

***Lexical***

The lexical level relates to the mental inventory of words: our vocabulary. From our vocabulary, words must be selected that convey the meaning of the preverbal message [25]. Several standard and psycholinguistic lexical variables can be derived from connected language. Lexical diversity in language output, for instance measured by *Type-Token Ratio* (TTR), is a standard measure reflecting the lexical richness of a language sample. TTR is calculated by dividing the number of unique nouns/verbs in a language sample by the total number of words. It is a widely used measure in language research in clinical populations but its sensitivity to sample size should be taken into account [37]. *Lexical accuracy*, on the other hand, expresses whether an utterance contains lexical errors such as semantically related substitutions (e.g., ‘tree’ instead of ‘plant’), unrelated substitutions (e.g., ‘bike’ instead of ‘plant’), or general all-purpose (GAP) verbs (e.g., ‘to *get* an ice-cream’ instead of the more specific ‘to *buy* an ice-cream’). Lexical accuracy has been shown to be a good predictor of language impairment in children [24,38–40].

In addition to these standard measures, there are psycholinguistic variables that reflect lexical difficulty, such as *age of acquisition* (AoA)*.* AoA refers to the age at which a word is acquired. The word ‘mom’, for instance, is typically acquired much earlier than the word ‘matrix’ [41]. A lower AoA has been found to facilitate lexical retrieval of nouns and verbs [9]. Further, people with aphasia may retain words learned earlier in life better than words learned later in life [12]. Another indicator of lexical complexity is *word* *frequency,* which refers to how often a word appears in language corpora and generally reflects its usage in everyday language [42]. Word frequency seems to have a facilitating effect on language processing. High-frequency words are easier to name [9,19] and understand [13] in people with aphasia, and were named faster in children [43]. An increased use of words learned earlier in life and that are more frequent might reflect difficulties in lexical retrieval, resulting in greater dependence on words that are more easily accessible in the mental lexicon.

***Morphosyntax***

Morphosyntax is the rule-based system which governs the internal structure of words (morphology) and sentences (syntax) [44], establishing the legal combinations (i.e., grammatical). The process of transforming ideas to grammatically correct sentences is referred to as ‘grammatical encoding’ [25,45]. The ease of grammatical encoding is influenced by morphosyntactic complexity, as reflected in several standard language sample measures. One of the most commonly used measures is *Mean Length of Utterance* (MLU), a rough measure of syntactic complexity. A shorter MLU is a well-established marker of impairment, for example in children with DLD [46], and adults with aphasia [47]. Furthermore, *grammatical accuracy* was found to be a very consistent marker of differences between typically developing children and children with DLD [48]. Another standard measure is the *finiteness index*, capturing the accurate production of finite verbs. A finite verb is for instance ‘walks’ in ‘the man walks’, as the verb form matches the third person singular of the subject, as opposed to the verb in ‘the man walking’. Producing finite verbs has been proven to be particularly difficult in children with DLD and can discriminate them from typically developing children [49,50]. When grammatical accuracy and finiteness were combined, they also appeared to detect difficulties in performance in children treated for a PFT [24].

In addition to these standard measures, several verb properties may reflect grammatical complexity. *Verb transitivity* expresses whether a verb requires an object or not. Transitive verbs, such as ‘to buy’, always require an object in the sentence (i.e., something that is bought). Intransitive verbs, such as ‘to dance’, do not require such an object. The addition of the object in transitive sentences adds to the grammatical complexity of a verb and its corresponding sentence, rendering intransitive sentences easier for children with language disorders [2,51], although an opposite pattern has also been reported in people with aphasia [10]. *Unaccusativity*, on the other hand, relates specifically to intransitive verbs, which can be divided into two subtypes: unergative and unaccusative verbs. For unergative verbs, the subject of the sentence (e.g., the man) is considered an agent (e.g., the man dances), that is, an entity which actively and volitionally performs the action. The subject of unaccusative verbs, on the other hand, (e.g., ‘the ice-cream’ or ‘the man’ in the following examples) is experiencing or undergoing the action (e.g., ‘the ice-cream melts’ or ‘the man faints’). In English, sentences with unergative and unaccusative verbs differ in difficulty, because the most common or ‘canonical’ sentence order places an agent in subject position before the verb [52]. Sentences with unaccusative verbs are thus considered to be non-canonical and have been shown to be difficult for children with DLD, who frequently tend to omit the subjects in unaccusative verb constructions [4]. A similar pattern was found in people with aphasia [10]. Identifying atypical usage of unaccusative versus unergative verbs could detect difficulties with syntactic processing.

Another property reflecting the complexity of verbs is their *regularity*, which indicates whether a verb follows a predictable pattern of inflection. In English, for instance, regular verbs form the third-person singular in the present tense by adding -s (e.g., ‘he walks’, ‘she talks’) and form the past tense by adding -(e)d (e.g., ‘he walked’, ‘she talked’) without changing the root of the verb. Irregular verbs, on the other hand, do not follow this pattern and often change the root of the verb when inflected. Examples are the verb ‘to have’, which is ‘he has’ in the third-person singular in the present tense, or the verb ‘to drink’, which is ‘drank’ in the past tense. Children with DLD and adults with aphasia have greater difficulty producing irregular verbs [6,14,18].

The aforementioned measures all reflect aspects of encoding at the morphosyntactic level of language and are essential for formulating sentences that conform to grammatical conventions. Errors in the sentence construction and atypical use of verb properties (e.g., more intransitive, unergative and irregular verbs) could be an indication of morphosyntactic difficulties.

***Phonology***

Phonology relates to the system and patterns of speech sounds that a language is composed of. At this level, a phonological plan is constructed that specifies how speech sounds and syllables are organized to produce fluent, intelligible speech [25]. A standard way to assess phonological skills is to examine the presence of *phonological errors*, such as substitutions, omissions, or consonant cluster reductions. These errors may indicate incomplete or inaccurate phonological representations [53,54].

Phonological properties of words, such as syllable structure, number of syllables, or overall word length, can reflect the level of phonological complexity a child can successfully produce [55]. For instance, speech units with a higher complexity, such as consonant clusters (e.g., string) and long words, require the coordination of more phonological elements [56] and place greater processing demands on phonological working memory [57], which makes them more prone to errors than simpler structures. The accuracy of consonant cluster production, reflected in the current study by the measure *cluster index*, is a commonly used measure in children with phonological impairments [58], and DLD [59].

A frequently examined property reflecting phonological complexity is *word length*, which can be defined as the number of syllables, letters, or phonemes in a word. For instance, *dog* is short, whereas *crocodile* is comparatively long. Studies into the effect of word length show a better performance on shorter words in children with DLD [5] and people with aphasia [20] and children treated for a PFT [24]. These patterns align with the word-length hypothesis of Baddeley et al. [57], which proposes that longer words place greater demands on phonological working memory, as more phonological material must be planned and maintained before articulation.

However, an inverse word-length effect has also been reported in aphasia, where shorter words pose more difficulty than longer ones [11,17]. Nonetheless, this pattern might not reflect phonological complexity per se but rather lexical-phonological competition: short words often have many phonological neighbors (i.e.,  words that sound similar and compete during retrieval), whereas longer words typically have fewer competitors. For example, *cat* has several neighbors (e.g., *rat*, *bat*, *cap*), whereas *elephant* does not. Such patterns may indicate difficulties at the interface of lexical selection, rather than a deficit in phonological complexity itself.

**References**

1. Abel AD, Rice ML, Bontempo DE. Effects of Verb Familiarity on Finiteness Marking in Children With Specific Language Impairment. J Speech Lang Hear Res JSLHR. 2015;58:360–72. https://doi.org/10.1044/2015_JSLHR-L-14-0003

2. Andreu L, Sanz-Torrent M, Legaz LB, MacWhinney B. Effect of verb argument structure on picture naming in children with and without specific language impairment (SLI). Int J Lang Commun Disord. 2012;47:637–53. https://doi.org/10.1111/j.1460-6984.2012.00170.x

3. Friedmann N, Novogrodsky R. Is the movement deficit in syntactic SLI related to traces or to thematic role transfer? Brain Lang. 2007;101:50–63. https://doi.org/10.1016/j.bandl.2006.09.006

4. Grela BG, Leonard LB. The use of subject arguments by children with specific language impairment. Clin Linguist Phon. Taylor & Francis; 1997;11:443–53. https://doi.org/10.3109/02699209708985206

5. Jackson E, Leitao S, Claessen M, Boyes M. Fast mapping short and long words: Examining the influence of phonological short-term memory and receptive vocabulary in children with developmental language disorder. J Commun Disord. 2019;79:11–23. https://doi.org/10.1016/j.jcomdis.2019.02.001

6. Jacobson PF, Yu YH. Changes in English Past Tense Use by Bilingual School-Age Children With and Without Developmental Language Disorder. J Speech Lang Hear Res. 2018;61:2532–46. https://doi.org/10.1044/2018_JSLHR-L-17-0044

7. Kambanaros M. Does verb type affect action naming in specific language impairment (SLI)? Evidence from instrumentality and name relation. J Neurolinguistics. 2013;26:160–77. https://doi.org/10.1016/j.jneuroling.2012.07.003

8. McMillen S, Anaya JB, Peña ED, Bedore LM, Barquin E. That’s hard! Item difficulty and word characteristics for bilinguals with and without developmental language disorder. Int J Biling Educ Biling. Routledge; 2022;25:1838–56. https://doi.org/10.1080/13670050.2020.1832039

9. Bastiaanse R, Wieling M, Wolthuis N. The role of frequency in the retrieval of nouns and verbs in aphasia. Aphasiology. 2016;30:1221–39. https://doi.org/10.1080/02687038.2015.1100709

10. Bastiaanse R, Van Zonneveld R. Sentence production with verbs of alternating transitivity in agrammatic Broca’s aphasia. J Neurolinguistics. 2005;18:57–66. https://doi.org/10.1016/j.jneuroling.2004.11.006

11. Best W. A Reverse Length Effect in Dysphasic Naming: When Elephant is Easier than Ant. Cortex. 1995;31:637–52. https://doi.org/10.1016/S0010-9452(13)80017-2

12. Brysbaert M, Ellis AW. Aphasia and age of acquisition: are early-learned words more resilient? Aphasiology. Routledge; 2016;30:1240–63. https://doi.org/10.1080/02687038.2015.1106439

13. DeDe G. Effects of Word Frequency and Modality on Sentence Comprehension Impairments in People with Aphasia. Am J Speech-Lang Pathol Am Speech-Lang-Hear Assoc. 2012;21:S103–14. https://doi.org/10.1044/1058-0360(2012/11-0082)

14. Ruth de Diego B, Costa A, Sebastián-Galles N, Juncadella M, Caramazza A. Regular and irregular morphology and its relationship with agrammatism: Evidence from two Spanish–Catalan bilinguals. Brain Lang. 2004;91:212–22. https://doi.org/10.1016/j.bandl.2004.02.007

15. Hirsh KW, Funnell E. Those old, familiar things: age of acquisition, familiarity and lexical access in progressive aphasia. J Neurolinguistics. 1995;9:23–32. https://doi.org/10.1016/0911-6044(95)00003-8

16. Jonkers R, Bastiaanse R. Action naming in anomic aphasic speakers: Effects of instrumentality and name relation. Brain Lang. 2007;102:262–72. https://doi.org/10.1016/j.bandl.2007.01.002

17. Lambon Ralph MA, and Howard D. Gogi Aphasia or Semantic Dementia? Simulating and Assessing Poor Verbal Comprehension in a Case of Progressive Fluent Aphasia. Cogn Neuropsychol. Routledge; 2000;17:437–65. https://doi.org/10.1080/026432900410784

18. Miozzo M. On the processing of regular and irregular forms of verbs and nouns: evidence from neuropsychology. Cognition. 2003;87:101–27. https://doi.org/10.1016/s0010-0277(02)00200-7

19. Nozari N, Kittredge AK, Dell GS, Schwartz MF. Naming and repetition in aphasia: Steps, routes, and frequency effects. J Mem Lang. 2010;63:541–59. https://doi.org/10.1016/j.jml.2010.08.001

20. Pashek GV, Tompkins CA. Context and word class influences on lexical retrieval in aphasia. Aphasiology. Routledge; 2002;16:261–86. https://doi.org/10.1080/02687040143000573

21. Milburn E, Warren T, Dickey MW. Idiom comprehension in aphasia: Literal interference and abstract representation. J Neurolinguistics. 2018;47:16–36. https://doi.org/10.1016/j.jneuroling.2018.02.002

22. Alyahya RSW, Halai AD, Conroy P, Lambon Ralph MA. Content word production during discourse in aphasia: Deficits in word quantity, not lexical-semantic complexity. J Cogn Neurosci. 2021;33:2494–511. https://doi.org/10.1162/jocn_a_01772

23. Chéileachair FN, Chondrogianni V, Sorace A, Paradis J, Aguiar VD. Developmental language disorder in sequential bilinguals: Characterising word properties in spontaneous speech. J Child Lang. 2023;50:954–80. https://doi.org/10.1017/S0305000922000241

24. Svaldi C, Paquier P, Keulen S, Van Elp H, Catsman-Berrevoets C, Kingma A, et al. Characterising the Long-Term Language Impairments of Children Following Cerebellar Tumour Surgery by Extracting Psycholinguistic Properties from Spontaneous Language. The Cerebellum [Internet]. 2023 [cited 2024 Jan 2]; https://doi.org/10.1007/s12311-023-01563-z

25. Levelt WJM, editor. Speaking: From Intention to Articulation [Internet]. Cambridge, MA: The MIT Press; 1989. https://doi.org/10.7551/mitpress/6393.003.0009

26. Brysbaert M, Warriner AB, Kuperman V. Concreteness ratings for 40 thousand generally known English word lemmas. Behav Res Methods. 2014;46:904–11. https://doi.org/10.3758/s13428-013-0403-5

27. Schwanenflugel PJ, Harnishfeger KK, Stowe RW. Context availability and lexical decisions for abstract and concrete words. J Mem Lang. 1988;27:499–520. https://doi.org/10.1016/0749-596X(88)90022-8

28. Goh WD, Yap MJ, Lau MC, Ng MMR, Tan L-C. Semantic Richness Effects in Spoken Word Recognition: A Lexical Decision and Semantic Categorization Megastudy. Front Psychol [Internet]. Frontiers; 2016 [cited 2025 Feb 3];7. https://doi.org/10.3389/fpsyg.2016.00976

29. Nickels L, Howard D. Aphasic naming: What matters? Neuropsychologia. 1995;33:1281–303. https://doi.org/10.1016/0028-3932(95)00102-9

30. Bonner MF, Vesely L, Price C, Anderson C, Richmond L, Farag C, et al. Reversal of the concreteness effect in semantic dementia. Cogn Neuropsychol. 2009;26:568–79. https://doi.org/10.1080/02643290903512305

31. Yi H-A, Moore P, Grossman M. Reversal of the concreteness effect for verbs in patients with semantic dementia. Neuropsychology. American Psychological Association; 2007;21:9–19. https://doi.org/10.1037/0894-4105.21.1.9

32. Scott GG, Keitel A, Becirspahic M, Yao B, Sereno SC. The Glasgow Norms: Ratings of 5,500 words on nine scales. Behav Res Methods. 2019;51:1258–70. https://doi.org/10.3758/s13428-018-1099-3

33. Altarriba J, Bauer LM, Benvenuto C. Concreteness, context availability, and imageability ratings and word associations for abstract, concrete, and emotion words. Behav Res Methods Instrum Comput J Psychon Soc Inc. 1999;31:578–602. https://doi.org/10.3758/bf03200738

34. Lin KR, Wisman Weil L, Thurm A, Lord C, Luyster RJ. Word imageability is associated with expressive vocabulary in children with autism spectrum disorder. Autism Dev Lang Impair. SAGE Publications Ltd; 2022;7:23969415221085827. https://doi.org/10.1177/23969415221085827

35. Svaldi C, Kohnen S, Robidoux S, Vos K, Reinders A, Arunachalam S, et al. Spoken verb learning in children with language disorder. J Exp Child Psychol. 2024;242:105881. https://doi.org/10.1016/j.jecp.2024.105881

36. He AX, Kon M, Arunachalam S. Linguistic context in verb learning: Less is sometimes more. Lang Learn Dev Off J Soc Lang Dev. 2020;16:22–42. https://doi.org/10.1080/15475441.2019.1676751

37. Hess CW, Sefton KM, Landry RG. Sample Size and Type-Token Ratios for Oral Language of Preschool Children. J Speech Lang Hear Res. American Speech-Language-Hearing Association; 1986;29:129–34. https://doi.org/10.1044/jshr.2901.129

38. Charest M, Skoczylas MJ. Lexical Diversity Versus Lexical Error in the Language Transcripts of Children With Developmental Language Disorder: Different Conclusions About Lexical Ability. Am J Speech Lang Pathol. American Speech-Language-Hearing Association; 2019;28:1275–82. https://doi.org/10.1044/2019_AJSLP-18-0143

39. Rice ML, Bode JV. GAPS in the verb lexicons of children with specific language impairment. First Lang. SAGE Publications Ltd; 1993;13:113–31. https://doi.org/10.1177/014272379301303707

40. Svaldi C, Galli J-I, Paquier P, Keulen S, Van Elp H, Catsman-Berrevoets C, et al. Errors in the Spontaneous Language of Survivors of Pediatric Cerebellar Tumors. The Cerebellum. 2025;24:1–7. https://doi.org/10.1007/s12311-024-01754-2

41. Kuperman V, Stadthagen-Gonzalez H, Brysbaert M. Age-of-acquisition ratings for 30,000 English words. Behav Res Methods. 2012;44:978–90. https://doi.org/10.3758/s13428-012-0210-4

42. Keuleers E, Brysbaert M, New B. SUBTLEX-NL: A new measure for Dutch word frequency based on film subtitles. Behav Res Methods. 2010;42:643–50. https://doi.org/10.3758/BRM.42.3.643

43. Alario F-X, Ferrand L, Laganaro M, New B, Frauenfelder UH, Segui J. Predictors of picture naming speed. Behav Res Methods Instrum Comput. 2004;36:140–55. https://doi.org/10.3758/BF03195559

44. Croft W. Morphosyntax: Constructions of the World’s Languages [Internet]. High. Educ. Camb. Univ. Press. Cambridge University Press; 2022 [cited 2025 Feb 3]. https://doi.org/10.1017/9781316145289

45. Slevc LR. Grammatical Encoding. In: Hartsuiker RJ, Strijkers K, editors. Lang Prod [Internet]. London: Routledge; 2023. 10.4324/9781003145790-2

46. Hewitt LE, Hammer CS, Yont KM, Tomblin JB. Language sampling for kindergarten children with and without SLI: mean length of utterance, IPSYN, and NDW. J Commun Disord. 2005;38:197–213. https://doi.org/10.1016/j.jcomdis.2004.10.002

47. Stark BC. A Comparison of Three Discourse Elicitation Methods in Aphasia and Age-Matched Adults: Implications for Language Assessment and Outcome. Am J Speech Lang Pathol. 2019;28:1067–83. https://doi.org/10.1044/2019_AJSLP-18-0265

48. Winters KL, Jasso J, Pustejovsky JE, Byrd CT. Investigating Narrative Performance in Children With Developmental Language Disorder: A Systematic Review and Meta-Analysis. J Speech Lang Hear Res. American Speech-Language-Hearing Association; 2022;65:3908–29. https://doi.org/10.1044/2022_JSLHR-22-00017

49. Bedore LM, Leonard LB. Specific Language Impairment and Grammatical Morphology. J Speech Lang Hear Res. American Speech-Language-Hearing Association; 1998;41:1185–92. https://doi.org/10.1044/jslhr.4105.1185

50. Leonard LB, Miller C, Gerber E. Grammatical Morphology and the Lexicon in Children With Specific Language Impairment. J Speech Lang Hear Res. American Speech-Language-Hearing Association; 1999;42:678–89. https://doi.org/10.1044/jslhr.4203.678

51. Pizzioli F, Schelstraete M-A. Children with specific language impairment: The effect of argument-structure complexity on auditory sentence comprehension. Clin Linguist Phon. Taylor & Francis Ltd; 2011;25:1–22. https://doi.org/10.3109/02699206.2010.509842

52. McAllister T, Bachrach A, Waters G, Michaud J, Caplan D. Production and comprehension of unaccusatives in aphasia. Aphasiology. 2009;23:989–1004. https://doi.org/10.1080/02687030802669518

53. Roepke E, Brosseau-Lapré F. Speech Error Variability and Phonological Awareness in Preschoolers. Am J Speech Lang Pathol. 2023;32:246–63. https://doi.org/10.1044/2022_AJSLP-22-00031

54. Preston J, Edwards ML. Phonological Awareness and Types of Sound Errors in Preschoolers With Speech Sound Disorders. J Speech Lang Hear Res. American Speech-Language-Hearing Association; 2010;53:44–60. https://doi.org/10.1044/1092-4388(2009/09-0021)

55. Stoel-Gammon C. The Word Complexity Measure: Description and application to developmental phonology and disorders. Clin Linguist Phon. Taylor & Francis Ltd; 2010;24:271–82. https://doi.org/10.3109/02699200903581059

56. Nam H, Goldstein L, Saltzman E. Self-organization of syllable structure: a coupled oscillator model. In: Pellegrino F, Marsico E, Chitoran I, Coupé C, editors. Approaches Phonol Complex [Internet]. Walter de Gruyter; 2009 [cited 2025 Jan 28]. p. 297–328. https://doi.org/10.1515/9783110223958.297

57. Baddeley AD, Thomson N, Buchanan M. Word length and the structure of short-term memory. J Verbal Learn Verbal Behav. 1975;14:575–89. https://doi.org/10.1016/S0022-5371(75)80045-4

58. McLeod S, Doorn J, Reed VA. Realizations of consonant clusters by children with phonological impairment. Clin Linguist Phon. 1997;11:85–113. https://doi.org/10.1080/02699209708985185

59. Scheffer A, Keij B, Hakvoort B, Ottow-Henning E, Gerrits E, Wijnen F. Speech sound development of young Dutch children with a developmental language disorder: A complex matter. Int J Lang Commun Disord. 2024;59:2706–22. https://doi.org/10.1111/1460-6984.13109
